# Universal Vector Calibration for Orientation-Invariant 3D Sensor Data †

**DOI:** 10.3390/s25154609

**Published:** 2025-07-25

**Authors:** Wonjoon Son, Lynn Choi

**Affiliations:** School of Electrical Engineering, Korea University, Seoul 02841, Republic of Korea; swj8905@korea.ac.kr

**Keywords:** vector calibration, three-dimensional sensor, coordinate system, indoor positioning, geomagnetic field, step detection

## Abstract

Modern electronic devices such as smartphones, wearable devices, and robots typically integrate three-dimensional sensors to track the device’s movement in the 3D space. However, sensor measurements in three-dimensional vectors are highly sensitive to device orientation since a slight change in the device’s tilt or heading can change the vector values. To avoid complications, applications using these sensors often use only the magnitude of the vector, as in geomagnetic-based indoor positioning, or assume fixed device holding postures such as holding a smartphone in portrait mode only. However, using only the magnitude of the vector loses the directional information, while ad hoc posture assumptions work under controlled laboratory conditions but often fail in real-world scenarios. To resolve these problems, we propose a universal vector calibration algorithm that enables consistent three-dimensional vector measurements for the same physical activity, regardless of device orientation. The algorithm works in two stages. First, it transforms vector values in local coordinates to those in global coordinates by calibrating device tilting using pitch and roll angles computed from the initial vector values. Second, it additionally transforms vector values from the global coordinate to a reference coordinate when the target coordinate is different from the global coordinate by correcting yaw rotation to align with application-specific reference coordinate systems. We evaluated our algorithm on geomagnetic field-based indoor positioning and bidirectional step detection. For indoor positioning, our vector calibration achieved an 83.6% reduction in mismatches between sampled magnetic vectors and magnetic field map vectors and reduced the LSTM-based positioning error from 31.14 m to 0.66 m. For bidirectional step detection, the proposed algorithm with vector calibration improved step detection accuracy from 67.63% to 99.25% and forward/backward classification from 65.54% to 100% across various device orientations.

## 1. Introduction

Modern devices such as smartphones, wearable devices, robots, and drones incorporate three-dimensional accelerometers, magnetometers, and gyroscopes, which enable the estimation of device’s movement, heading, speed, and posture. Various applications using these 3D sensors are gaining attention in the smartphone and robot markets. These include PDR (Pedestrian Dead Reckoning) [1], posture estimation [2], gesture recognition [3], activity classification [4], fall detection [5], and augmented-reality overlay alignment [6].

To ensure a reliable measurement with 3D sensors, the first critical step is often applying the general sensor calibration procedure to correct for intrinsic errors. This calibration primarily focuses on enhancing the measurement accuracy of the sensor itself. For example, various techniques are used to compensate for errors like hard and soft iron distortions in magnetometers [7,8,9,10,11], or biases and scale factors in accelerometers and gyroscopes [12,13].

While this traditional sensor calibration procedure targets reliable and accurate measurement with the sensor hardware itself, our vector calibration is a completely different process, targeting the sensor data transformation in the three-dimensional space, since sensor measurements in three-dimensional vectors are highly sensitive to device orientation. This means that the same motion can be recognized as a different motion when the smartphone’s tilt or heading direction is different.

**Figure 1:**
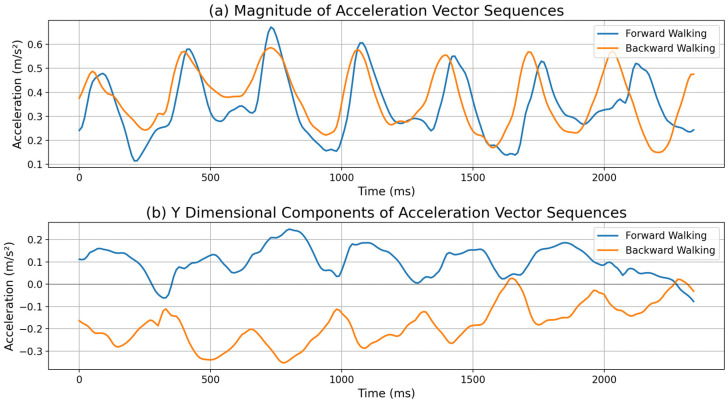
Comparison of accelerometer values during forward (blue line) and backward (orange line) walking. (**a**) The magnitudes of accelerometer vector sequences show similar values in both tests. (**b**) The y dimensional component values of acceleration vector sequences show a clear difference. Therefore, using the vector value enables not only step detection but also classification between forward and backward steps, as discussed in Section 5.

Most studies have addressed this problem by using the magnitude values of three-dimensional vectors [14] or taking ad hoc approaches such as assuming fixed device holding postures [15]. The magnitude of a three-dimensional vector remains constant regardless of device orientation. However, this approach loses the directional information of a motion by converting a three-dimensional vector into a single scalar value. For example, as shown in Figure 1a, forward and backward walking may produce similar magnitude values of three-dimensional accelerometers despite having opposite directions. This can simplify algorithms for step counting. However, since the detailed interpretation of individual three-dimensional vector values is not possible, it cannot determine the exact direction of the movement. Figure 1b shows the acceleration vector values in the y dimension during forward and backward walking. During forward walking, the acceleration in the y dimension is positive, while during backward walking it is negative. By using the polarity difference in the sensor vector values it is possible to implement directional step counting rather than traditional step counting.

**Figure 2:**
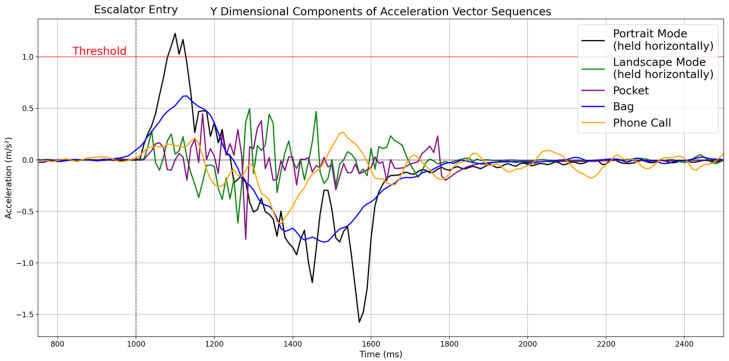
Acceleration vector values measured along the device’s y-axis during escalator entry for five different smartphone-carrying positions: hand-held in portrait mode (black), landscape mode (green), in pants pocket (purple), in a bag (blue), and during a phone call (orange). The red horizontal line denotes the 1.0 m/s^2^ detection threshold, and the dashed vertical line marks the moment of entry. Only the portrait posture exceeds the threshold, whereas the other carrying positions remain below it.

In addition, ad hoc posture assumptions work under controlled laboratory conditions but often fail in real-world scenarios. In the real world, users carry smartphones or wearable devices in various orientations while walking, making a phone call or carrying them in different positions such as pants pockets, shirt pockets, and bags. As an example, Figure 2 shows the acceleration vector values in the device’s y dimension when taking an escalator while carrying a smartphone in various positions. When the acceleration vector value exceeds the threshold (1.0 m/s^2^) marked in red, it is recognized as taking an escalator. When the user holds the smartphone horizontally in portrait mode, the vector value exceeds the threshold, and the algorithm recognizes the escalator usage (black line). However, when the user holds the smartphone in landscape mode (green line), carries it vertically in the front pocket of the pants with the top end facing down (purple line), carries it in a bag horizontally (blue line), and uses it for a phone call (orange line), the vector value does not exceed the threshold. This occurs because when taking the escalator, the device’s y-axis becomes misaligned with the user’s movement direction. Consequently, the algorithm fails to detect escalator usage even though the user is taking the escalator. This example demonstrates how sensor orientation-dependent algorithms that perform well under controlled laboratory conditions could fail in real-world scenarios.

To process a sensor’s three-dimensional vector data regardless of device orientation, we propose a universal vector calibration algorithm that enables consistent three-dimensional vector data measurements for the same physical activity, independent of how users hold or rotate their devices.

**Figure 3:**
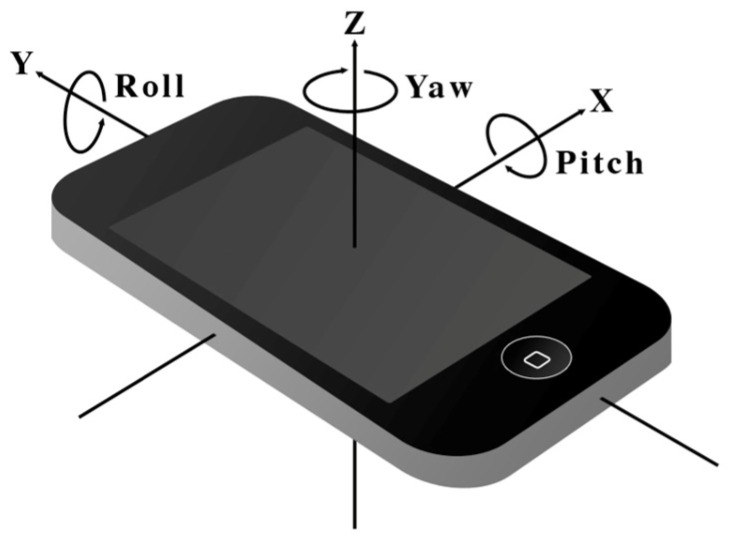
Roll, pitch, and yaw rotation on a smartphone. This coordinate system is defined such that the XY plane is horizontal to the ground, and the Z-axis is aligned with gravity [18].

The proposed algorithm consists of two steps. First, we calibrate the device’s tilting using a measured pitch and roll angle. As shown in Figure 3, pitch angle represents the rotation angle along the x-axis, and roll angle represents the rotation angle along the y-axis. Second, we calibrate the device’s yaw rotation, which is the rotation along the z-axis, to align with a reference coordinate system. This reference coordinate system varies depending on the application requirements. It may be a user coordinate system aligned with the user’s eye direction, a global coordinate system aligned with Earth’s gravity and the user’s moving direction, or an absolute coordinate system aligned with an absolute orientation. We discuss the types of coordinate systems for various applications in the following section. Then, we demonstrate how we apply the vector calibration algorithm to two different applications: a geomagnetic field-based indoor positioning system [16,17] and bidirectional step detection with forward and backward walking.

## 2. Classification of Coordinate Systems

Before we introduce our vector calibration algorithm, we first define various categories of coordinate systems used in sensor-based applications. Table 1 shows the types of coordinate systems and the corresponding application examples where each coordinate system is used. We categorize coordinate systems into two main types: relative coordinate systems and absolute coordinate systems. A coordinate system is *relative* when its coordinate is defined along with an object’s orientation, such as human body orientation or device orientation. In contrast, a coordinate system is *absolute* when all three axes are aligned to a specific orientation in the three-dimensional space regardless of device rotation or user movement. However, since any object in a space, including Earth, is moving continuously relative to another, it is very difficult to define an absolute coordinate system. Therefore, we define a coordinate system as absolute when its coordinates are aligned, with Earth’s gravity direction being negative in the z dimension while its x-y plane is in parallel to the Earth’s surface. An example of an absolute coordinate system is one where its positive x coordinates point East while its positive y coordinates point North.

Table 1 shows three examples of relative coordinate systems. First, the local coordinate system is physically aligned with a device’s orientation [19]. As shown in Figure 3, the x-, y-, and z-axes of the coordinate system are aligned with the smartphone’s internal sensor orientations. Second, the *user* coordinate system is physically aligned with user orientation, where the user’s head becomes the positive z dimension and the user’s eye level defines the xy plane. The direction of the user’s eye becomes the positive y dimension while their left-hand side becomes the negative x dimension. This coordinate system rotates when the user turns their head or changes posture so that it follows the user’s viewing direction. Third, the global coordinate system is an example of a user coordinate system, with the xy plane aligned horizontally to the ground and perpendicular to Earth’s gravity. This coordinate system maintains horizontality regardless of how the user tilts or moves but allows rotation only along the z-axis depending on the user’s movement direction. This is generally called the global coordinate system [12].

Depending on the application requirements, different reference coordinate systems are employed. For example, geomagnetic field-based indoor positioning [16,17] requires an absolute coordinate system aligned with the magnetic field map collection direction, since magnetic fingerprints are collected in a fixed orientation and real-time positioning accuracy depends on aligning three-dimensional magnetic field vectors to this reference orientation. Bidirectional step detection requires the global coordinate system because forward and backward walking are defined relative to the user’s moving direction, rather than to the device orientation. When a user holds a smartphone in various orientations such as portrait and landscape modes or carries the device in their pocket and in a bag, the same physical walking motion may generate different acceleration patterns in the local coordinate system. Therefore, the global coordinate system is essential to consistently distinguish movement directions across different device orientations. Smartwatch applications use the local coordinate system, as user interactions such as gestures and taps are naturally interpreted relative to the watch’s physical orientation on the wrist [20]. VR (Virtual Reality) applications require the user coordinate system since the application must respond to the user’s head and body movements while accommodating various gaming postures including standing, sitting, and lying down [21]. The LiDAR SLAM scanning application requires the global coordinate system to maintain consistent spatial mapping as the device moves through different orientations during environment scanning [22].

Our vector calibration algorithm transforms sensor measurements from the local coordinate system to any target reference coordinate system used in each application. For instance, geomagnetic field-based indoor positioning uses the absolute coordinate system defined along with the direction of the magnetic field map collection as a reference coordinate system. And bidirectional step detection uses the global coordinate system as a reference coordinate system. The following section presents the details of our universal vector calibration algorithm.

## 3. Vector Calibration Algorithm

A three-dimensional vector is used to measure the sensor readings in the local coordinate system. We denote the raw 3D sensor measurement by

$$v_{local}=\left[\begin{array}{c} v_{x}\\ v_{y}\\ v_{z} \end{array}\right]\;\;\;\;\in \;R^{3},$$

where $v_{x}$, $v_{y}$, and $v_{z}$ represent the measured values along the sensor’s x-, y-, and z-axes, respectively.

The proposed algorithm consists of two stages. First, we transform the vector $v_{local}$ from the local coordinate system to the global coordinate system by calibrating the device’s tilt. To calibrate tilting, we first estimate the device’s pitch (ϕ) and roll (θ) angle using the gravity vector computed from $v_{local,\;acc}$, which is the $v_{local}$ of the accelerometer sensor. The $v_{local,\;acc}$ measures not only linear acceleration caused by device movement but also the Earth’s gravitational acceleration (approximately 9.8 m/s^2^). Since gravity is always directed downward toward Earth’s center, the gravity vector computed from the accelerometer’s three axes indicates how the device is tilted relative to the vertical direction. We isolate the gravity vector by filtering out the high-frequency motion components from the raw accelerometer data, retaining only the low-frequency gravitational component [12]. More specifically, we employ an exponential smoothing filter [23], which is a type of low-pass filter particularly well-suited for real-time gravity estimation in mobile devices. Among the various moving average filters [24,25], we decided to use the exponential smoothing filter because it requires saving only a single previous sample, unlike other moving average filters [26] that require saving multiple previous samples. This results in O (1) memory complexity and minimal processing overhead, making it computationally efficient for real-time operation while providing optimal balance between noise reduction and responsiveness to orientation changes.

The filtered acceleration vector converges to the gravity vector $g={\left[g_{x},\;g_{y},g_{z}\right]}^{T}$, as the high-frequency linear acceleration components are progressively attenuated while the constant gravitational component is preserved.

The roll and pitch angles can be computed as

$$\phi =atan2(g_{y},\;g_{z}),\;\;\;\theta =atan2(-g_{x},\;\sqrt{{g_{y}}^{2}+{g_{z}}^{2}})$$

However, roll and pitch calculated in this way may contain errors. This is because the gravity component cannot be perfectly filtered from the raw accelerometer data. The accelerometer measures the total force acting on it and cannot distinguish between the gravitational force and the inertial forces caused by the device’s movement. Therefore, our low-pass filtering approach operates under the assumption that gravity is a constant, low-frequency signal, while forces from user motion are rapidly changing, high-frequency signals. This assumption is particularly effective during typical user activities such as walking, sitting, or jogging, where the user’s movement can change over a short period of time. In this environment the low-pass filter can effectively separate the gravity vector. However, in an environment where the user’s movement changes slowly and continuously, such as riding in a car or a train, the filter’s ability to separate the gravity vector can be reduced. The resulting error in the calculated pitch and roll angles is typically within ±1° under static conditions and ±3° during normal walking. In situations with slow and continuous acceleration, this error can temporarily increase up to ±10°.

The rotation matrices that correct the device’s tilt (pitch and roll) are defined as follows:

Rx−ϕ =  100 0cosϕsinϕ 0−sinϕcosϕ,

$$R_{y}(-\theta )\;=\;\left[\begin{array}{ccc} cos\theta & 0 & -sin\theta \\ 0 & 1 & 0\\ sin\theta & 0 & cos\theta \end{array}\right]$$

Here, $R_{x}(-\phi )$ represents rotation around the x-axis to eliminate pitch angle ϕ, and $R_{y}(-\theta )$ represents rotation around the y-axis to eliminate roll angle θ. The combined tilt correction rotation matrix is

$$R_{tilt}(\phi ,\;\theta )=R_{y}(-\theta ){\;R}_{x}(-\phi )$$

By applying this combined rotation matrix to the raw sensor vector, we obtain a tilt-corrected vector.

$$v_{tilt}=R_{tilt}(\phi ,\;\theta )v_{local}=R_{y}(-\theta ){\;R}_{x}(-\phi )\left[\begin{array}{c} v_{x}\\ v_{y}\\ v_{z} \end{array}\right]$$

At this point, the vector $v_{tilt}$ is independent of the device’s roll and pitch variations. The tilt correction ensures that consistent vector values are measured for the same physical activity regardless of whether the device is held upright, tilted sideways, and rotated forward or backward. However, when the device is rotated around the z-axis (yaw rotation), different vector values are still measured for the same physical activity.

Second, to correct yaw rotation, we transform the tilt-corrected vector to align with a target reference coordinate system. As described in Table 1, we select the appropriate reference coordinate system based on the specific application requirements. For instance, geomagnetic field-based indoor positioning requires alignment with the magnetic field map collection direction, while bidirectional step detection aligns with the user’s moving direction. The key principle is that we define a reference direction ($\psi_{ref}$) specific to each application context.

We first obtain the device’s yaw angle $\psi_{yaw}$ from the gyroscope, where $\psi_{yaw}$ denotes the rotation around the z-axis. We then determine the yaw correction angle Δψ as the difference between the measured yaw angle and the reference direction.

$$\Delta \psi =\psi_{yaw}-\psi_{ref}$$

From the Δψ, we can build the yaw correction rotation matrix

$$R_{z}\left(-\Delta \psi \right)\;=\;\left[\begin{array}{ccc} cos\Delta \psi & sin\Delta \psi & 0\\ -sin\Delta \psi & cos\Delta \psi & 0\\ 0 & 0 & 1 \end{array}\right],$$

and apply it to the tilt-corrected vector to obtain the fully calibrated vector.

$$v_{calib}=R_{z}\left(-\Delta \psi \right)\;v_{global}$$

$$=R_{z}(-\Delta \psi )R_{y}(-\theta ){\;R}_{x}(-\phi )\left[\begin{array}{c} v_{x}\\ v_{y}\\ v_{z} \end{array}\right]$$

Now this calibrated vector $v_{calib}$ maintains consistent measurements for the same physical activity regardless of device orientation, including pitch, roll, and yaw rotations.

The pseudo-code of the vector calibration algorithm is shown in Algorithm 1.

**Algorithm 1**: Pseudo-code of the vector calibration algorithm
**Input**: $v_{local}$: A raw 3D vector to be calibrated (e.g., magnetic field vector) $a_{raw}$: The raw 3D vector from the accelerometer $\psi_{yaw}$: The current yaw angle of the device $\psi_{ref}$: The application-specific reference yaw angle **Output**: $v_{calib}$ The calibrated 3D vector
**Constant**: α = 0.8(The smoothing coefficient)
**Persistent State**: $a_{filtered\_prev}$: The filtered acceleration vector from the previous time step1// Stage1: Pitch & Roll Correction2
$a_{filtered\_current}\;=\alpha \;*\;a_{filtered\_prev\;+\;}(1-\alpha )\;*\;a_{raw}$
3
$g=a_{filtered\_current}\;//\;g=\;{\left[g_{x},\;g_{y},g_{z}\right]}^{T}$
4
$a_{filtered\_prev}\;=\;a_{filtered\_current}$
5
θ = atan2(−gx,sqrt(gy^2+gz^2)) // roll angle
6
$\phi \;=\;atan2(g_{y},\;g_{z})\;//\;\mathrm{pitch angle}$
7
$R_{x}\;=\mathrm{rotation\_matrix\_x}(-\phi )$
8
$R_{y}\;=\mathrm{rotation\_matrix\_y}(-\theta )$
9
$v_{tilt}\;=\;R_{y}\;*\;R_{x}\;*\;v_{local}$
1011// Stage2: Yaw Correction12
$\Delta \psi \;=\;\psi_{yaw}-\psi_{ref}$
13
$R_{z}\;=\mathrm{rotation\_matrix\_z}(-\Delta \psi )$
14
$v_{calib}\;=\;R_{z}\;*\;v_{tilt}$
15
$\mathrm{return}\;v_{calib}$


The computational complexity of the algorithm is O (1) for each input vector. Each vector calibration involves three 3 × 3 rotation matrix constructions and multiplications, along with trigonometric computations for the rotation matrix elements. Since these operations are performed on constant-sized data structures (3 × 1 vectors and 3 × 3 matrices) regardless of the application scale or dataset size, the processing time remains constant per vector.

We measured the runtime performance of our vector calibration algorithm on a Samsung Galaxy S23 Plus smartphone with Android 14 (Samsung Electronics Co., Ltd., Suwon, Republic of Korea). The average execution time for calibrating a single three-dimensional vector is approximately 0.15 ms. This processing time is significantly lower than typical sensor sampling intervals in Android systems. According to Android 14 specifications, motion sensors are system-limited to specific maximum sampling rates: the accelerometer and the gyroscope operate at up to 200 Hz (5 ms intervals) while the magnetometer operates at up to 50 Hz (20 ms intervals). Our calibration processing time of 0.15 ms represents only 3% of the accelerometer and gyroscope sampling interval, and 0.75% of the magnetometer sampling interval. This substantial margin ensures real-time performance without affecting the overall system responsiveness, even when processing sensor data at maximum sampling rates.

## 4. Evaluation of Geomagnetic Field-Based Indoor Positioning

**Figure 4:**
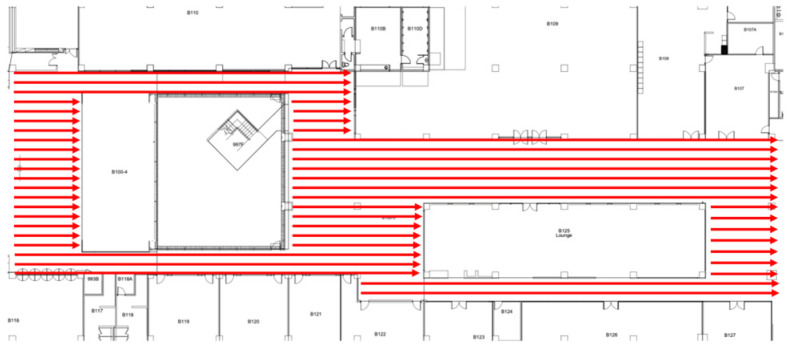
When we collected the magnetic vectors during the field map construction, we moved in the red arrows’ direction.

We first applied the proposed vector calibration algorithm to a geomagnetic field-based indoor positioning system (IPS) and evaluated its performance [27]. To build the IPS, we first collected a magnetic field map. We used the Hana Square underground facility in Korea University Science Campus as our IPS testbed, whose dimensions are 26 m × 95 m. We collected magnetic field data at every walkable location at 60 cm intervals while holding the smartphone horizontally and walking in a single, consistent direction, as shown in Figure 4.

**Figure 5:**
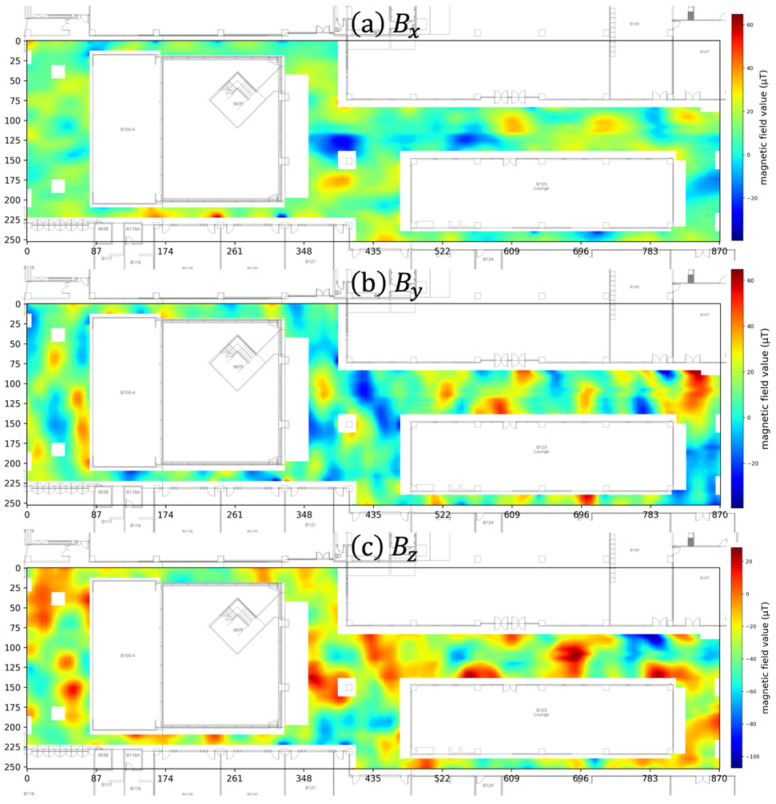
The three-dimensional magnetic field map of our Hana Square testbed, showing the individual vector components: (**a**) the x dimension ($B_{x}$), (**b**) the y dimension ($B_{y}$), and (**c**) the z dimension ($B_{z}$).

At each point, the smartphone’s magnetometer always recorded the three-dimensional magnetic field vector ($B_{x},\;B_{y},\;B_{z}$). We then generated a three-dimensional magnetic field vector map by spatially interpolating the collected three-dimensional magnetic field vector data. Figure 5 visualizes the magnetic field map. Specifically, Figure 5a is the map generated by interpolating the x component ($B_{x}$) of the vectors, Figure 5b shows the map for the y component ($B_{y}$), and Figure 5c shows the map for the z component ($B_{z}$).

To apply vector calibration in a real-time magnetic field-based IPS [16,17], the measured magnetic field vectors must be aligned with the magnetic field map collection direction. Therefore, we set the map collection direction shown in Figure 4 as the reference direction $\psi_{ref}$. When the user starts walking, we calculate $\Delta \psi \;=\;\psi_{yaw}-\psi_{ref}$ using the user’s moving direction $\psi_{yaw}$ estimated from compass and gyroscope data and apply this to calibrate the magnetic field vectors in real-time.

To evaluate the performance of our proposed vector calibration algorithm, we first analyzed the sequence matching improvement by comparing the raw and calibrated magnetic vectors against the ground truth map data. Subsequently, we also evaluated the improvement in localization accuracy by applying the calibration to an LSTM-based IPS [17]. A reliable ground truth data collection is essential for this evaluation, including the collection of the real-time magnetic vector sequences for the user’s test paths with varying orientations as well as the selection of the target vector sequences from the magnetic map.

**Figure 6:**
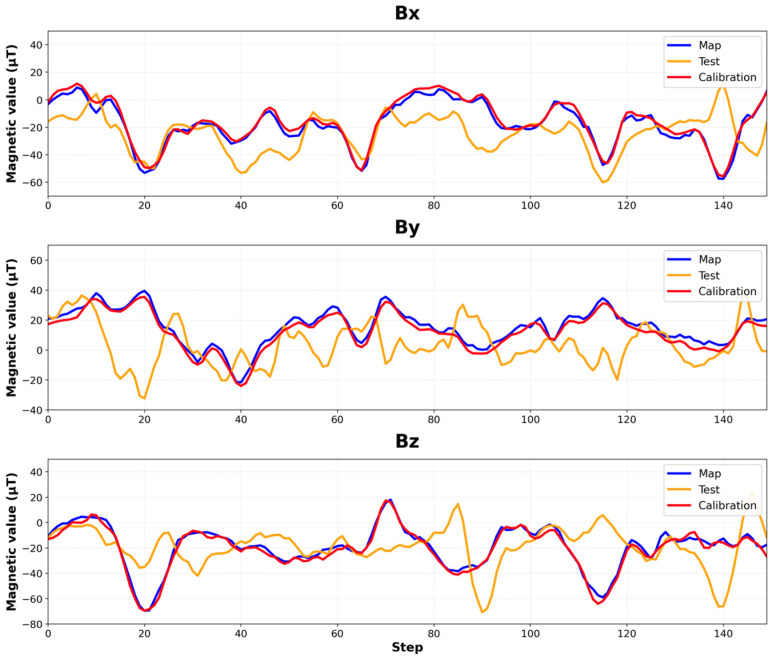
Magnetic vector sequences in the x, y, and z dimensions for one of our test paths. The blue line shows the sequence of values stored in the magnetic field map while the orange line shows the sequence of values sampled by the user during the test while walking on the same path with a random orientation. The red line shows the result after calibration.

To create this experimental setup, we use six different test paths with varying movement patterns to evaluate our calibration algorithm under different scenarios. Each test path was planned to include multiple directional changes relative to the magnetic field map collection direction. For example, one test path involved walking 30 steps in the same direction as the map collection direction (0°), then turning 90° clockwise and walking 50 steps, followed by another 90° clockwise turn and walking 50 steps, then a final 90° clockwise turn and walking the remaining 70 steps in the final direction (270°) relative to the map collection direction. These various directional changes allowed us to evaluate the calibration algorithm’s performance across various orientation differences between real-time movement and the original map collection direction. The ground truth map data sequences, as shown in Figure 6, consist of values extracted from the pre-collected magnetic field map at locations corresponding to these test paths. To minimize the errors caused by inter-device sensor biases, both map collection and test evaluations were conducted using the same smartphone.

Figure 6 compares the sequence of magnetic vectors in the x, y, and z dimensions in the magnetic field map against the sequences of magnetic vectors collected during a test case with and without vector calibration. The blue line shows the vector sequence of the magnetic field map. The orange line shows the vector sequence during the test without calibration. During the test, if the device’s orientation aligns with the magnetic field map collection direction, the vector sequence collected during the test generally matches well with the corresponding sequence in the magnetic field map. However, if the device orientations are different from each other or the smartphone’s tilting changes during the test, the vector values completely mismatch. In the figure, the average differences between the vectors in the magnetic field map and the collected vectors without vector calibration are 13.83 μT, 16.51 μT, and 16.90 μT in the x, y, and z dimensions, respectively. The maximum difference reaches as high as 71.7 μT. These large differences make indoor positioning impossible with raw vectors in the existing geomagnetic field-based IPS.

However, as shown by the red line in Figure 6, after applying our vector calibration the calibrated vector sequences now match well with the original sequence in the magnetic field map. The calibrated vectors consistently match regardless of device orientation or tilting changes. For six test cases, the average differences between the calibrated vector values and those in the magnetic field map are 2.42 μT, 2.84 μT, and 2.44 μT in the x, y, and z dimensions, respectively. This suggests that our algorithm effectively addresses the device orientation dependency of magnetic field vectors and can be applied for real-time positioning in magnetic field-based indoor positioning systems.

To demonstrate the practical benefits of our vector calibration, we evaluated its impact on a geomagnetic field-based IPS using recurrent neural network (RNN) models, including a Long Short-Term Memory (LSTM) model [17]. While basic RNNs can process sequence data, they often struggle with long-term dependencies, gradually forgetting information from earlier parts of a long sequence. LSTM models address this limitation through internal memory cells that enable them to retain and utilize information from extended sequences, making them a better choice for indoor positioning systems with long sequences. The primary objective of our evaluation is to demonstrate the effectiveness of vector calibration across different applications, rather than comparing the impact of deep neural network models on the IPS’s performance.

To train the LSTM model for supervised training, we first generated training data from the pre-collected magnetic field map. To create paths like actual pedestrian movements, we used a modified random waypoint model [28]. This model simulates walking patterns by selecting a random starting point within the testbed, moving a random distance in a random direction, and then changing the direction again. Through this process, we generate 400,000 random training paths, each of which consists of 100 steps. A total of 60% of the datasets are used for training, 20% for validation, and the remaining 20% for the test. The architecture of our LSTM model consists of three hidden layers, each containing 300 hidden nodes. This network is designed to process an input sequence of 100 three-dimensional magnetic vectors and to output a two-dimensional (x, y) location coordinate. The detailed hyperparameters for the model are summarized in Table 2.

After training, our geomagnetic field-based IPS using the LSTM model shows an average positioning error of 0.43 m for the test set. However, in real-time testing with a smartphone, the performance varies dramatically depending on how device orientation is handled. When using raw vectors without any calibration, the positioning error increases to 30.18 m. This severe degradation occurs because device orientation changes during user movement can affect the measured magnetic vector sequences, causing them to mismatch with the vector sequences in the pre-collected magnetic field map, resulting in inaccurate position estimates.

Using the vector magnitude sequences instead of raw vector sequences, which is the most common approach for handling orientation dependency, performed better with an average positioning error of 10.48 m. While the magnitude-based approach resolves device orientation dependency, resulting in better positioning performance than using uncalibrated vectors, the positioning error is still quite large. This limitation arises because this approach uses only a single scalar magnitude value instead of utilizing the full three-dimensional vector. Since the magnitude-based approach represents the current best practice for handling 3D sensor data in geomagnetic field-based indoor positioning, the existing geomagnetic field-based IPSs face inherent limitations in further improving positioning accuracy.

In contrast, by applying our vector calibration algorithm to 3D sensor data, we can use the entire 3D vector sequences for sequence matching in the LSTM model. We can reduce the average positioning error of our IPS to 0.66 m, which is a modest degradation compared to the training error of 0.43 m. Thus, with vector calibration, we can address the fundamental performance limitation of the existing geomagnetic field-based IPS by preserving the full three-dimensional vector information.

**Figure 7:**
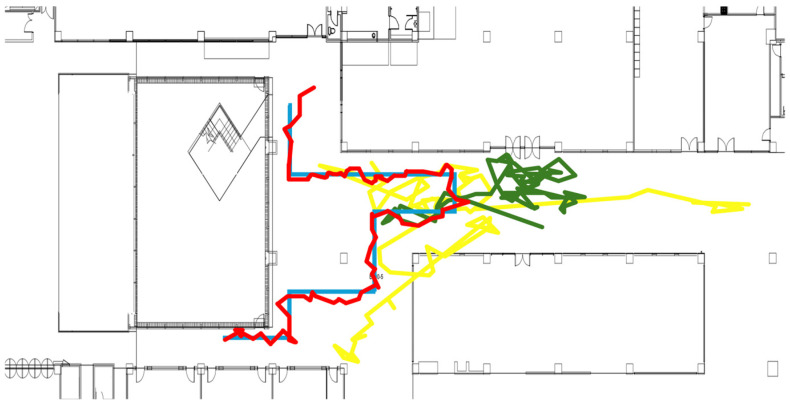
Positioning results of LSTM-based geomagnetic field IPS for a single test path. The blue line shows the actual test path, while the yellow, green, and red lines show the predicted paths generated by the LSTM model using vector magnitudes only, uncalibrated vectors, and calibrated vectors, respectively.

Figure 7 compares the test path with the paths predicted by the LSTM-based IPS for one of the six test paths. The blue line represents the ground truth path, while the other colored lines are paths predicted by the LSTM model using different vector processing approaches. The green line shows the path predicted by the LSTM model without calibration. As shown in the figure, the positioning results completely mismatch with the actual path. The yellow line shows the results of using vector magnitudes as the input to the LSTM model. As the figure illustrates, the magnitude approach produces better results than using the raw vectors without calibration. But it still exhibits significant positioning errors. However, with vector calibration, the LSTM model now accurately estimates user movement paths like the actual path, as shown in the red line. Table 3 summarizes the average positioning errors of the LSTM model using these three different vector processing approaches for six test paths.

## 5. Evaluation of Bidirectional Step Detection

**Figure 8:**
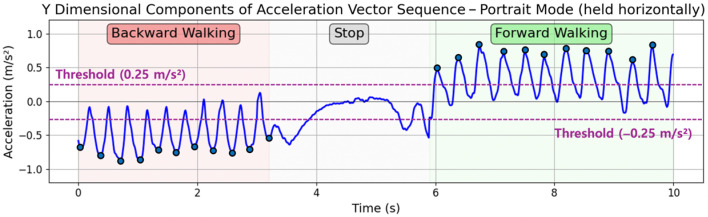
Bidirectional step detection results with smartphone held horizontal to the ground in portrait mode.

Our second target application is bidirectional step detection. So far traditional step detection algorithms [29,30] only count forward steps, missing backward steps and backward movement. We applied the proposed vector calibration algorithm to extend classical step detection to include bidirectional step detection. For bidirectional step detection, we use the acceleration vector in the y dimension. Since raw acceleration values contain noises that make analysis difficult, we apply a low-pass filter to the acceleration data. We detect both lower and upper peaks from the sampling data of the acceleration vector in the y dimension, recognizing a single peak in a sine curve as a single step. A forward step is detected when an upper peak exceeds a positive threshold of +0.25 m/s^2^, while a backward step is detected when a lower peak falls below a negative threshold of −0.25 m/s^2^. This is illustrated in Figure 8.

**Figure 9:**
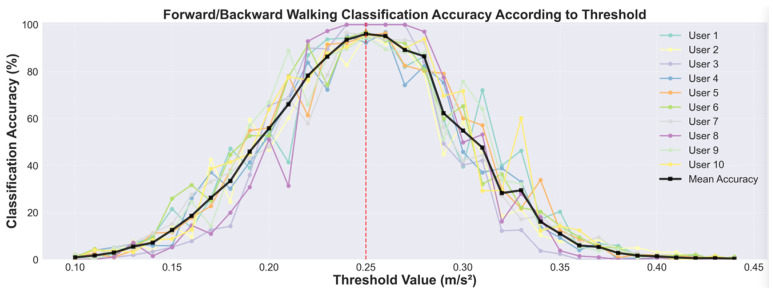
The accuracy of forward and backward step classification in terms of the acceleration vector in the y dimension. The graph shows the classification accuracy for 10 individual users (colored lines) and their means (black line) across a range of threshold values. The mean accuracy is maximized at 96.0% when the threshold is ±0.25 m/s^2^, indicated by the red dashed line.

The threshold was determined through experimental validation. We collected a dataset where 10 users of varying gender, age, and height were instructed to walk 100 steps forward and then 100 steps backward with an approximate step length of 60 cm. We then evaluated the forward and backward walking classification accuracy as we varied the threshold value from ±0.1 to ±0.45 m/s^2^. As shown in Figure 9, the average classification accuracy across all users peaked when the threshold was set to ±0.25 m/s^2^. Therefore, we select this threshold value for our bidirectional step detection.

Figure 8 shows the test results when a user walks backward, stops, and then walks forward while holding a smartphone horizontal to the ground in portrait mode. When the smartphone is held in this orientation, the smartphone’s y-axis aligns with the user’s moving direction, enabling accurate step detection and bidirectional classification without vector calibration. The blue dots marked on the line graph indicate the detected upper peaks for forward steps and lower peaks for backward steps. In this test example, all detected lower peaks during backward walking fall below the −0.25 m/s^2^ threshold and all detected upper peaks during forward walking exceed the +0.25 m/s^2^ threshold, showing that the suggested threshold successfully distinguishes between forward and backward steps.

To apply vector calibration to bidirectional step detection, we set the reference direction $\psi_{ref}$ to the user’s moving direction. We assume that the user’s moving direction matches with the device’s positive y-axis in its local coordinate when a user holds a smartphone in portrait mode. However, depending on the user’s grip posture, the heading of the smartphone’s y dimension may not match with the user’s moving direction, making it difficult to accurately estimate the user’s moving direction with only the smartphone’s sensors. To solve this problem, we use the IPS to help determine the user’s moving direction regardless of the grip posture. By analyzing the sequence of previous coordinates from the IPS, we can estimate the user’s moving direction. We then calculate the difference between this moving direction and the device’s orientation using the gyroscope ($\Delta \psi \;=\;\psi_{yaw}-\psi_{ref}$) and apply this to align the acceleration vectors with the user’s moving direction regardless of how the smartphone is rotated. This alignment enables an accurate distinction between forward and backward walking based on the calibrated acceleration vector.

**Figure 10:**
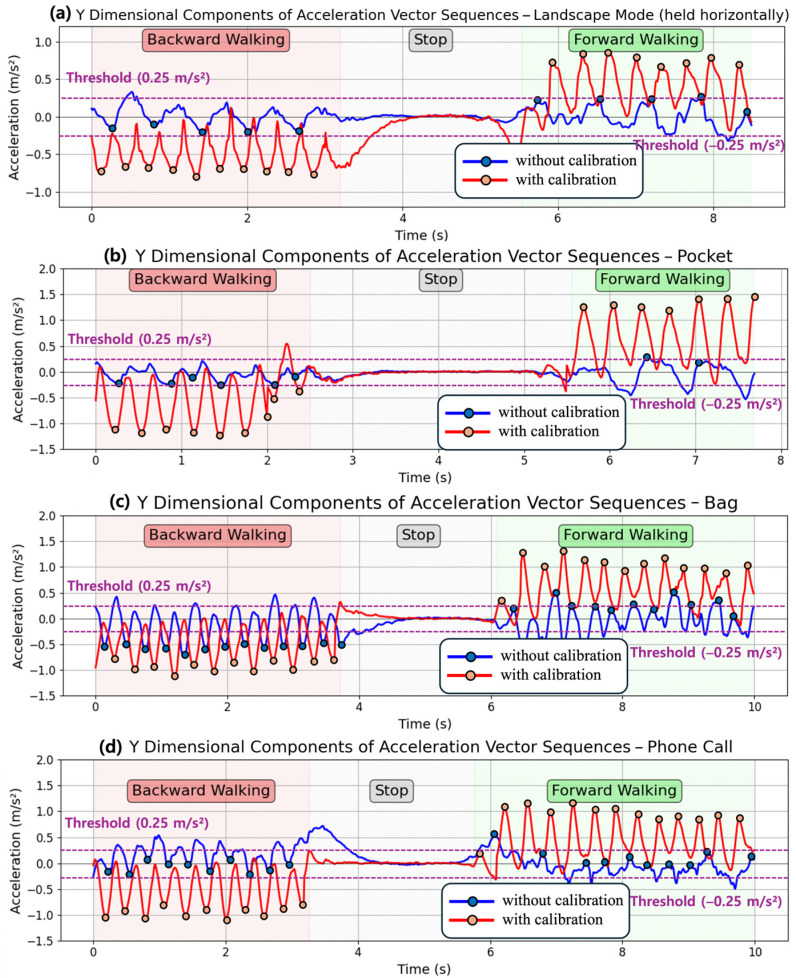
Comparison of bidirectional step detection performance when a smartphone is carried in different orientations and positions with and without vector calibration.

Figure 10 shows the bidirectional step detection results when the smartphone is carried in various orientations and in different positions: (a) held horizontally in landscape mode, (b) carried in their pants pocket, (c) carried in a backpack, and (d) held during a phone call. In contrast to the multi-user experiment shown in Figure 9, the experiment shown in Figure 10 was conducted with only a single user. Unlike the case in Figure 8, when the smartphone’s y-axis is not aligned with the user’s moving direction, the acceleration vector is measured according to the device’s orientation rather than the user’s actual moving direction. The blue lines show the results without vector calibration, where the acceleration patterns vary depending on device orientation, making it difficult to detect steps accurately and to distinguish between forward and backward walking. In Figure 10a,b, while the user took 19 and 16 steps during walking, the number of detected peaks was 10 and 8, resulting in step detection accuracies of 52% and 50%. Moreover, in Figure 10c,d, bidirectional classification fails with rates of 52% and 81%, respectively, due to peaks exceeding the threshold during backward walking or remaining below the threshold during forward walking.

With vector calibration, the bidirectional step detection performance improves, as shown in the red lines in Figure 10, by aligning the acceleration vector to the user’s moving direction. Regardless of device orientation, the calibrated acceleration data reflects the user’s actual movement rather than the device’s orientation, enabling reliable step detection and accurate forward and backward walking classification. Table 4 summarizes the performance of step detection and forward/backward step classification over 200 steps. When comparing the results with and without calibration, step detection accuracy improved from an average of 67.63% to 99.25%, and forward/backward walking classification improved from an average of 65.54% to 100%.

## 6. Conclusions

In this paper, we proposed a universal vector calibration algorithm that addressed the fundamental challenge of orientation dependency in three-dimensional sensor data. The algorithm enables consistent vector measurements for the same physical activity regardless of device orientation, overcoming the limitations of existing magnitude-based and ad hoc posture assumption approaches.

Our calibration algorithm works in two stages. First, we correct device tilting using pitch and roll angles computed from raw 3D accelerometer vector data. Then, we calibrate yaw rotation to align with application-specific reference coordinate systems. We systematically categorize coordination systems into relative (local, user, global) and absolute types, providing a framework for selecting appropriate references based on application requirements.

Evaluation of two 3D sensor applications demonstrates the effectiveness of our vector calibration algorithm. For geomagnetic field-based indoor positioning, the calibration algorithm achieved an 83% reduction in vector mismatches between sampled magnetic vectors and reference magnetic field map vectors across all three dimensions. Additionally, it reduced the average positioning error from 30.18 m to 0.66 m in the LSTM-based IPS. For bidirectional step detection, the algorithm dramatically improved step detection accuracy from 67.63% to 99.25% and forward/backward classification accuracy from 65.54% to 100% across various device orientations and carrying positions.

The proposed universal calibration algorithm provides 3D sensor applications with consistent and reliable performance regardless of how users naturally hold or carry their devices in real-world scenarios. Beyond the evaluated applications, our calibration framework can be readily applied to various three-dimensional sensor-based applications. For smartwatch gesture recognition, the algorithm enables consistent gesture detection regardless of wrist rotation or watch orientation, improving recognition accuracy across different hand positions. In XR gaming applications, our calibration allows for seamless gameplay across diverse user postures by automatically aligning sensor measurements with the user’s orientation, eliminating manual recalibration needs. For LiDAR SLAM applications, the calibration ensures consistent spatial mapping regardless of device orientation during environment scanning, resulting in more robust localization and mapping performance.

## Figures and Tables

**Table 1:** Coordinate system types, their characteristics, and the corresponding application examples.

Coordinate System Type		Reference Orientation	Free Axes	Characteristics	Application Examples
Relative	Local	Device hardware	All three axes	· Physically fixed to device hardware · Axes rotate with device	· Double tab or wrist rotation detection on smartwatch
	User	User’s eye direction	All three axes	· Aligns with user’s eye direction · Axes rotate with user’s head tilt/orientation	· XR gaming with various postures · Head tracking in XR devices
	Global	Earth’s gravity and user’s movement direction	Z-axis only	· xy plane aligned horizontally to ground · Free rotation along the z-axis only	· Bidirectional step detection (forward/ backward walking) · Three-dimensional scanning with LiDAR SLAM
Absolute		An absolute orientation in 3D space	None	· All three axes aligned to a specific orientation in 3D space · No free rotation along any axis	· Geomagnetic-based indoor positioning

**Table 2:** Hyperparameters of our LSTM model.

Hyperparameter	Value
Hidden nodes	600
Hidden layers	3
Batch size	300
Learning rate	0.0005
Loss function	Mean squared error
Optimizer	Adam
Activation function	tanh/ReLU
Train/validation/test	60%:20%:20%
Epochs	500
Path length	100

**Table 3:** LSTM positioning errors for six test paths using different vector processing approaches.

	Magnitude-Based Approach	Without Vector Calibration	With Vector Calibration
Test 1	8.841 m	17.842 m	0.881 m
Test 2	9.873 m	21.301 m	0.407 m
Test 3	11.249 m	52.485 m	0.716 m
Test 4	4.230 m	33.147 m	0.553 m
Test 5	8.421 m	42.127 m	0.562 m
Test 6	18.242 m	19.958 m	0.866 m
Average	10.143 m	31.143 m	0.664 m

**Table 4:** Bidirectional step detection accuracy when a smartphone is carried in various orientations and positions.

	Step Detection		Forward/Backward Step Classification	
	WithoutCalibration	WithCalibration	WithoutCalibration	WithCalibration
Landscape mode	53% (106/200)	100% (200/200)	79.2% (84/106)	100% (200/200)
In pants pocket	42.5% (85/200)	98.5% (197/200)	81.2% (69/85)	100% (197/197)
In a backpack	86.5% (173/200)	99% (198/200)	52.02% (90/173)	100% (198/198)
During a phone call	88.5% (177/200)	99.5% (199/200)	49.72% (88/177)	100% (177/177)

## Data Availability

Data are contained within the article.
